# How Well Does a Sequential Minimal Optimization Model Perform in Predicting Medicine Prices for Procurement System?

**DOI:** 10.3390/ijerph18115523

**Published:** 2021-05-21

**Authors:** Amarawan Pentrakan, Cheng-Chia Yang, Wing-Keung Wong

**Affiliations:** 1 Department of Healthcare Administration, Asia University, Taichung 41354, Taiwan; 108211019@live.asia.edu.tw (A.P.); chengchia@asia.edu.tw (C.-C.Y.); 2 Department of Pharmacy Administration, Faculty of Pharmaceutical Sciences, Prince of Songkla University, Songkhla 90112, Thailand; 3 Fintech Center, and Big Data Research Center, Department of Finance, Asia University, Taichung 41354, Taiwan; 4 Department of Medical Research, China Medical University Hospital, Taichung 40447, Taiwan; 5 Department of Economics and Finance, The Hang Seng University of Hong Kong, Hong Kong 999077, Hong Kong

**Keywords:** sequential minimal optimization, prediction model, feature selection, medicine price

## Abstract

The lack of an efficient approach in managing pharmaceutical prices in the procurement system led to a substantial burden on government budgets. In Thailand, although the reference price policy was implemented to contain the drug expenditure, there have been some challenges with the price dispersion of medicines and pricing information transparency. This phenomenon calls for the development of a potential algorithm to estimate appropriate prices for medical products. To serve this purpose, in this paper, we first developed the model by the sequential minimal optimization (SMO) algorithm for predicting the range of the prices for each medicine, using the Waikato environment for knowledge analysis software, and applying feature selection techniques also to examine improving predictive accuracy. We used the dataset comprised of 2424 records listed on the procurement system in Thailand from January to March 2019 in the application and used a 10-fold cross-validation test to validate the model. The results demonstrated that the model derived by the SMO algorithm with the gain ratio selection method provided good performance at an accuracy of approximately 92.62%, with high sensitivity and precision. Additionally, we found that the model can distinguish the differences in the prices of medicines in the pharmaceutical market by using eight major features—the segmented buyers, the generic product groups, trade product names, procurement methods, dosage forms, pack sizes, manufacturers, and total purchase budgets—that provided the highest predictive accuracy. Our findings are useful to health policymakers who could employ our proposed model in monitoring the situation of medicine prices and providing feedback directly to suggest the best possible price for hospital purchasing managers based on the feature inputs in their procurement system.

## 1. Introduction

The cost of pharmaceutical products is one of a considerable proportion of total healthcare expenditure in many countries, resulting in between 20% and 60% [1]. Additionally, achieving a data analysis technique for purchasing medicine remains a challenge in the pharmaceutical procurement system because the market is inefficient. In the pharmaceutical market, many economic market structures could face imperfect competition and become a challenge to control the price of medicines, for instance, in monopoly and oligopoly markets. In both cases, they can be significant barriers to entry for other firms. Furthermore, drug pricing can vary for many features, such as the differences in formulations, designs, packaging, or sale volumes. Some buyers might pay above the lowest available price for the same product because of information asymmetry, single vendor, inelastic demand, and among other reasons [2]. Due to these complexities, it is difficult to determine the appropriate intervention for managing the prices of medicines. One of the typical requirements to control medicine prices of the Food and Drug Administration in many countries is that identical pharmaceutical products should have little price dispersion; however, in the current market, the products of various brands typically have seen the dispersion of pricing [3]. Pharmaceutical procurement in differing environments was classified into different price ranges [4].

The limitations of procurement procedures in the pharmaceutical market could be different from country to country. In Thailand, pharmaceutical procurement is a complex system that relates to several agencies, organizations, and manufacturers. The pharmaceutical procurement data are gathered to estimate a reference price of each medicine. The reference price is determined as the maximum price that hospitals can procure their product. However, this strategy has not been successful in controlling the price dispersion for pharmaceuticals which have been routinely encountered [5]. As described by Ngorsuraches and Chayakan [6], although the reference price policy is a relatively simple tool used in drug procurement, there are some limitations in pricing information transparency. There are many features associated with pricing in the pharmaceutical market in Thailand, such as the characteristics of medicine products, the conditions of both the local and international dealing, and the bargaining powers of different buyers. More specifically, the inadequate monitoring process and the lack of access to sufficient information and technology can result in purchasing unnecessarily high prices and also lead to facilitate improper influence on the procurement procedure by special interest groups [7]. Among hospitals in Thailand, identical medical products were procured at different prices, even buying from the same vendor and distributor [8]. One of the important reasons is that using a reference price method is still difficult for identifying the same medicine exactly due to the difference in trade names, manufacturers, strength, dosage form, and packaging. These factors have a significant impact on how well users can effectively select the target price on the various combination features. Furthermore, the hospitals are still limited to understand the current market price. This engenders difficulty in calculating the optimal price of their product. Therefore, policymakers still need to find effective measures or new tools which have high separating power and high sensitivity to monitor the situation of each drug price. Moreover, determining the range of the prices over setting the reference prices should be considered because it can develop human interpretation and be compatible with heterogeneous data [9].

Accordingly, a model that can effectively predict the range of the prices for each medicine should be explored. The choice between the statistical and machine learning (ML) methods may seem fuzzy but it can be considered on the primary objective [10]. In some medical data, the relationships of variables are reasonably straightforward, for example, those between diabetes risk and body mass index. This can be well presented using relatively simple models of statistical inference to understand the relationships between factors. Conversely, in pharmaceutical data, the prices of medicines can vary according to several factors, and many relationships could not reasonably explain, for instance, those between packaging and price setting. In this case, the ML technique might be a good choice as the relationship between several inputs that are complex and usually non-linear. The primary consideration in the ML field is an accurate prediction. The individual features and the outcome can have a little relationship if the prediction is accurate. More specifically, a technique of sequential minimal optimization (SMO) [11] which is an effective algorithm in support vector machine learning, is widely used to classify several datasets based on the features given [12].

To assist policymakers to monitor the prices of medicines, in this study, we focused on investigating the performance of the model of the SMO algorithm for predicting the range of the prices for each medicine. We expected that our findings can be useful to health policymakers who could employ this model as an application in monitoring the situation of medicine prices and providing feedback directly to suggest the best possible price for hospital purchasing managers based on their feature inputs in the procurement system.

## 2. Literature Review

### 2.1. Pharmaceutical Procurement

Pharmaceutical procurement represents a considerable part of the expenditure of the healthcare sector. It is important for operational performance [13]. The main aspect of procurement in all countries is the same, that is, to make available the right medicines of assured quality in the right quantities from reliable suppliers at the lowest possible prices [1]. The appropriate procurement processes have been considered essential to develop competition between drug suppliers through the transparent selection process to develop the system efficiency by enhancing economies of scale. In public procurement, governments are responsible to guarantee that procurement is carried out efficiently, the quality of public service delivery, and protecting the public interest. Additionally, the government and decision-makers have usually relied on empirical procurement data to identify effective patterns, test theories, and analyze the medication use, cost, and price in pharmaceuticals. Poor processing in procurement often creates an easy target for corruption. Pharmaceutical procurement is even more susceptible to corruption than conducting in other sectors [14]. This is because drug procurement, involving many influential factors such as the practices in determining the brand of medicine or sale volume, is often subjective; the monitoring quality standards in drug procurement of each unit level are difficult; suppliers set different prices for the same medicine product and can make unreasonable prices; some marketing strategies by pharmaceutical companies try to convince demand for their products; and another challenge is presented by emergency conditions, which call for the acute intervention.

One of the policies used to control drug expenditure in many countries is price regulation policy through reference prices. Reference drug pricing can be applied to different levels of medicine groups [15], as drugs can involve in the same Anatomical Therapeutic Chemical (ATC) classification system group. This policy can have various evidence of its impact. In European countries and Canada, for example, they can save a lot of government budget from this intervention [16]. Similarly, in Colombia, the price regulation policy through reference prices represented in reducing approximately 41% in the medicine prices [17]. Despite this revealed positive outcome, the reference price strategy has led to a reduction of transparency and accessibility of medicine price information such as pharmaceutical suppliers try to prevent buyers from knowing that the same medicine products are sold at a lower price in another area but claim that same price [18]. Accordingly, price dispersion in the same type of medicine has been encountered. Furthermore, using different procurement methods also has an impact on the purchase prices of medicines, for example, using centralized drug procurement has significantly associated with a reduction of pharmaceutical prices [19] when comparing with a decentralized system managed by local levels. However, the procurement structures also depend on the country’s economy. Currently, with the integration of information technology and new algorithms, developing an efficient application that can build up an information circle between the government procurement data center and hospital purchasing managers would be useful for managing medicine prices.

### 2.2. John Platt’s Sequential Minimal Optimization (SMO)

While there have been various implementations of support vector machine (SVM) algorithms in the past years [20,21], in this study, we focus on the sequential minimal optimization (SMO) algorithm, an effective algorithm in SVM learning introduced by Platt [11]. The key feature of the SMO algorithm is that it iteratively selects subsets only of size two to put chunking to the extreme and then optimize the target function with features given. This algorithm can solve analytically better than other support vector learnings because it does not require using a quadratic optimizer. SMO has greatly performed on scaling for all datasets. This technique is simple, dependable, and easy to be used. In comparison, the speed of executing the SMO algorithm is much faster than all SVM algorithms [22].

The SMO algorithm has been used in many fields [12,23,24] and demonstrated to get very good performance with sparse data inputs, even which imbalanced data, because it requires much shorter kernel computation time. It has also been demonstrated to have a high predictive capability to find out optimal values and unknown patterns. For example, Pham et al. [23] have demonstrated that the SMO algorithm outperforms both vote feature intervals and logistic regression on landslide prediction. Sunarya et al. [25] have shown that the SMO algorithm has higher predictive accuracy than the principal component analysis method in predicting commodity prices. Additionally, Ince and Trafalis [26] have found that the SMO algorithm outperforms the multi-layer perceptron networks in terms of the mean square error evaluation on the stock price prediction. The SMO is a state-of-the-art technique for classification but, as far as we know, it has not been applied for the pharmaceutical sector that consists of complex feature inputs of products, and its processes are related to several agencies, organizations, types of medicines, and manufacturers. In this work, we aimed to investigate the performance of the model of the SMO algorithm on predicting the prices of each medicine.

## 3. Data, Variables, and Methodology

### 3.1. Data and Variables

According to the recent literature, there are several factors related to the pricing of each medicine [27]. In the pharmaceutical markets, the price of the same medicine can be different among hospitals in different regions [28]. The distributions of the prices of drugs depend on the characteristics of medical products including the type of medicine, trade name, dosage form, and package [29]. Additionally, procurement methods could have an impact on purchase prices. For example, using public bidding practices can drive down the procurement prices of medicines [30]. On the other hand, using specific selection methods without competition could reduce the power of negotiating lower prices. We exhibit all the relevant features that could influence the medicine pricing in Table 1. The study datasets comprised pharmaceutical procurement data in Thailand from January to March 2019. The dataset contained 2424 records without missing values. We hypothesized that eight features (the segmented buyers, the generic product groups, trade product names, procurement methods, dosage forms, pack sizes, manufacturers, and total purchase budgets) could be used in developing the model to predict the prices for each medicine effectively. These features will be examined by using the feature selection method, a process of selecting a subset of relevant features for the classifier.

**Figure 1:**
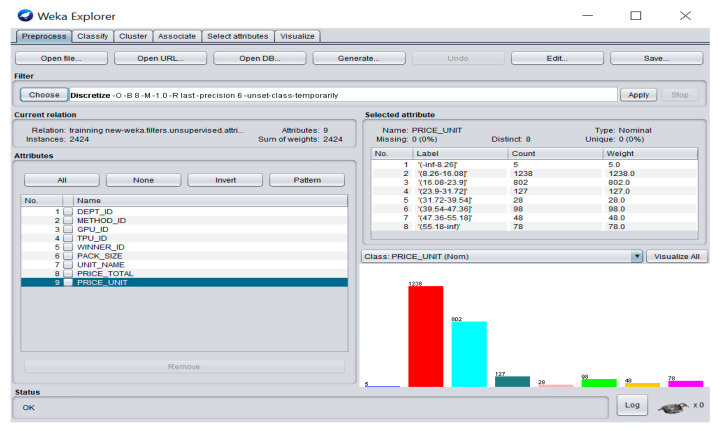
Pre-processing stage and discretization in WEKA with eight class labels.

We now define all the variables used in our paper. The output variable used in our paper is PRICE which is the price per unit of medicine that discretized into eight class labels of price ranges for medicine, as shown in Figure 1: “(−inf–8.26]” for medicine product pricing less than or equal 8.26; “(8.26–16.08]” for medicine product pricing interval from more than 8.26 to 16.08; “(16.08–23.9]” for medicine product pricing interval from more than 16.08 to 23.90; “(23.9–31.72]” for medicine product pricing interval from more than 23.90 to 31.72; “(31.72–39.54]” for medicine product pricing interval from more than 31.72 to 39.54; “(39.54–47.36]” for medicine product pricing interval from more than 39.54 to 47.36; “(47.36–55.18]” for medicine product pricing interval from more than 47.36 to 55.18; and “(55.18–inf)” for medicine product pricing more than 55.18.

We use other eight procurement features as the input variables, including DEPT, GPU, TPU, METHOD, UNIT, SIZE, WINNER, and TOTAL. DEPT is variable of purchasing department that consists of thirteen departments to make the decision to purchase medicines for hospitals, GPU is the name of the generic product used in this study under Anatomical Therapeutic Chemical Classification (ATC) code A02BC01 (omeprazole, parenteral form) which involves two different labels: omeprazole 40 mg and omeprazole 20 mg, TPU is the trade product name that includes twenty-two different names, METHOD is the procurement method that includes two different methods: bidding method and specific selection method, UNIT is the dosage form that includes two different forms, SIZE is the pack size that has seven different sizes; WINNER is the variable of winning supplier that consists of six different firms who are selected to sell the medical product to hospitals, and TOTAL is the total purchase budget that discretized into eighty-four different labels. These eight features are used in developing the model to predict the price ranges for medicine.

### 3.2. Conceptual Framework

**Figure 2:**
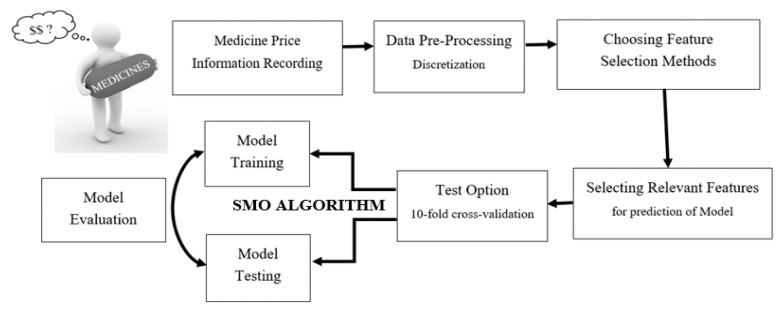
Summarized conceptual framework and research procedure.

The study procedures are listed in the conceptual framework exhibited in Figure 2.

In this study, we use the software of the Waikato environment for knowledge analysis (WEKA, University of Waikato, Hamilton, New Zealand) [31] which is an open-source Java-based software, and is facilitated the implementation of several machine learning algorithms, including sequential minimal optimization (SMO) algorithm. SMO in WEKA is dependable and has shown good performance for either binary or non-binary input data [32]. WEKA is a user-friendly software supporting various data mining tasks, including data preprocessing, classification, feature selection, and visualization. We employ the data preprocessing function in WEKA for discretizing the price data to the range of the prices before developing the model to reduce a large amount of data and reduce the levels of data complexity [9]. We then develop the model by using SMO for predicting the prices of medicines and applying feature selection techniques to obtain improving predictive accuracy.

### 3.3. Classifier

Sequential minimal optimization (SMO) is developed by employing the principle that the original quadratic programming problem produced when using the support vector machines (SVM) algorithm that can be decomposed into a series of the smallest possible sub-problems [11]. In our study, we suppose (*x*, *y*) is a vector of training dataset such that during the training process, a nonlinear classification problem with a dataset $\left\{\left(x_{i},\;y_{i}\right)\right\}\;$in which $x_{i}$ is the *i*th input pattern of the selected input variables (the segmented buyers, the generic product groups, trade product names, procurement methods, dosage forms, pack sizes, manufacturers, or total purchase budgets), and $y_{i}$ = {i=1, 2, ···, 8} is a class label of the output variable (as shown in Figure 1), where there are eight class labels for the range of drug prices; for example, $y_{i}$ = 1 means $x_{i}$ is in class 1. This multiclass classification problem is normally solved by decomposition to several binary problems for which the standard SVM can be used. However, as described by Platt [11], a quadratic programming problem, where the objective function *Q* depends on a set of Lagrange multipliers α = {$\alpha_{i}\}\;$as shown in the following maximization problem:

(1) $$Max\;Q\left(\alpha \right)=\sum_{i=1}^{n}\alpha_{i}-\frac{1}{2}\sum_{i=1}^{n}\sum_{j=1}^{n}y_{i}y_{j}\alpha_{i}\alpha_{j}k\left(x_{i},x_{j}\right)\;$$

subject to:

(2) $$0\leq \alpha_{i}\leq C,\;for\;i=1,\;2,\ldots ,\;n$$

(3) $$\overset{n}{\underset{i=1}{\sum }}\alpha_{i}y_{i}=0$$

in which α = {$\alpha_{i}\}\;$denotes a set of Lagrange multipliers of the sample, C represents the hyperparameter of SVM that manages the trade-off between allowance and maximizing margin for misclassification, and $k\left(x_{i},x_{j}\right)$ is a kernel function [33]. This problem described above can be solved by using the SMO algorithm. When choosing a pair of multipliers $\alpha_{1}$ and $\alpha_{2}$, we reduce the constraints to be:

(4) $$0\leq \alpha_{1},\;\alpha_{2}\leq \;C$$

Considering the Lagrange multipliers $\alpha_{1}$ and $\alpha_{2}$ to be optimized and keeping all other multipliers (i=3, 4,…, n) to be constants, we rewrite Equation (3) to be:

(5) $$y_{1}\alpha_{1}+y_{2}\alpha_{2}=-\overset{n}{\underset{i=3}{\sum }}\alpha_{i}y_{i}$$

Replacing $-\sum_{i=3}^{n}\alpha_{i}y_{i}$ by a constant *k*, Equation (5) becomes

(6) $$y_{1}\alpha_{1}+y_{2}\alpha_{2}=constant\;\left(k\right)$$

The SMO first optimizes the quadratic programming problem by determining a Lagrange multiplier $\alpha_{1}$. The optimal value of $\alpha_{1}$ is obtained by finding $\alpha_{1}^{new,unclipped}$ by restricting it with the following upper bound *U* and lower bound *L* limits, as mentioned in Platt [33]:

(7) $$\alpha_{1}=\{\begin{array}{c} U\;if\;\alpha_{1}^{new,unclipped}>U\\ \alpha_{1}^{new,unclipped}\;if\;L<\alpha_{1}^{new,unclipped}\leq U\;\\ L\;if\;\alpha_{1}^{new,unclipped}\leq \;L\; \end{array}.$$

A similar procedure can be used to find other optimal Lagrange multipliers $\alpha_{i}$ for i=2, 3, 4,…, n. The selection of these variables is guided by some heuristics, including choosing the variable with the maximum step size $\Delta \alpha_{n}=\alpha_{n}^{new}-\alpha_{n}^{old}$. This iterative process is repeated until it converges and the Karush–Kuhn–Tucker (KKT) conditions [33] are satisfied by all $\alpha_{1},\;\alpha_{2},\ldots ,\;\alpha_{n}$ variables. In this situation, the objective function is minimized.

**Figure 3:**
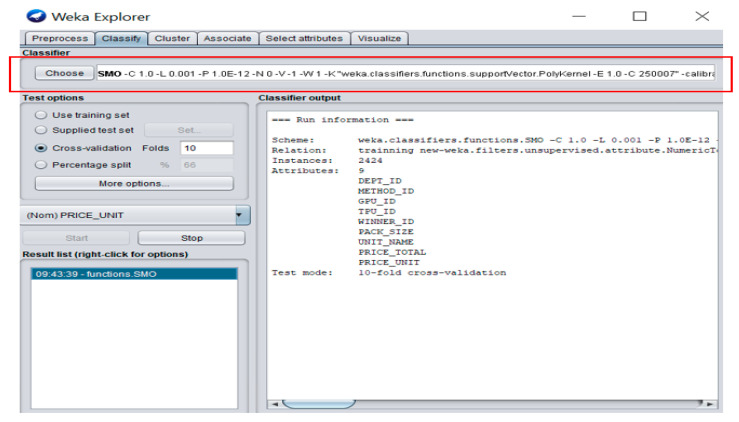
Sequential minimal optimization classifier in WEKA Software.

In the study, we employ the SMO classifier implemented in the Waikato environment for knowledge analysis (WEKA) software, as shown in Figure 3. The 10-fold cross-validation is used as a test option, and the test result shows that the model provides the highest accuracy when the value of the punishment factor (C) is 1, the value of the tolerance parameter (L) is 0.001, roundoff error is set to 1.0E−12, and the polynomial kernel is chosen as the kernel function of SMO.

However, the SMO algorithm still cannot handle large amounts of irrelevant features. To circumvent the limitation, efficient feature selection, the process of selecting a subset of essential features for the algorithm used, is recommended to circumventing the difficulty.

### 3.4. Feature Selection

In our analysis, we assumed that eight features, namely, the segmented buyers, the generic product groups, trade product names, procurement methods, dosage forms, pack sizes, manufacturers, and total purchase budgets can be used in developing the model to predict the prices for each medicine effectively. These features were examined by using the feature selection method [34], the process to remove all irrelevant features and choose only important features associated with the prediction of the prices for each medicine. We expected that drugs grouped in the same set of such feature inputs should be classified together and then used to validate the classification of drug prices. Roobaert et al. [35] indicated that feature selection can help the generalization performance of support vector learning techniques. Currently, several feature selection methods have been developed and implemented in the Waikato environment for knowledge analysis software. We applied the following four widely-used feature selection techniques: correlation-based feature subset selection (CFS), gain ratio, information gain, and wrapper subset evaluation in our study. The significant features selected by these methods were identified and compared to determine the solution with the best predictive accuracy. Among these feature selection methods, we hypothesized that using a sequential minimal optimization algorithm with a suitable feature selection method could improve the predictive accuracy.

### 3.5. Test Options

This study applied the option of 10-fold cross-validation, which was widely adopted to test the prediction accuracy and error of a model. Cross-validation has been extensively used for estimating the performances of different classification models [21,36]. In this approach, given data were split into 10-fold sub-samples with similar sample sizes and distributions. Nine of the subsets were applied to train the model, and the remaining subset was applied to test the model. This process was repeated ten times—that is to say, every subset was used as the test set once. This task was implemented in the Waikato environment for knowledge analysis software and it was easy to use.

### 3.6. Model Evaluation Metrics

After the model was developed and validated by a 10-fold cross-validation test, we can obtain feedback from metrics that can explain the performance of the model. Generally, accuracy constitutes the traditional metric for performance evaluation and comparison among different prediction models; accuracy can be expressed as the difference between the predicted class and the true class of data [37]. However, using accuracy alone to evaluate algorithms under conditions of imbalanced distribution of class conditions might produce misleading outcomes because algorithms are highly biased toward the majority classes. Accordingly, this study evaluated model performance by using several relevant metrics [20], including accuracy rate, precision, recall (sensitivity), F-measure, and an area under the receiver operating characteristic curve (ROC) area. We also calculated the Kappa coefficient that was a measure of the percentage of agreement and confusion matrix that revealed the number of correct and incorrect predictions for each class. In this process, we hypothesized that the sequential minimal optimization model could have good performance for predicting medicine prices.

## 4. Empirical Analysis

This section discusses the empirical analysis of our study. We first present the descriptive statistics of variables used in this study. We then discuss the results of the feature analysis, followed by discussing the performance analysis, including accuracy, precision, recall, F-measure, receiver operating characteristic (ROC) area, and the Kappa coefficient. We also represent the results of the confusion matrix in this section.

### 4.1. Descriptive Statistics

**Figure 4:**
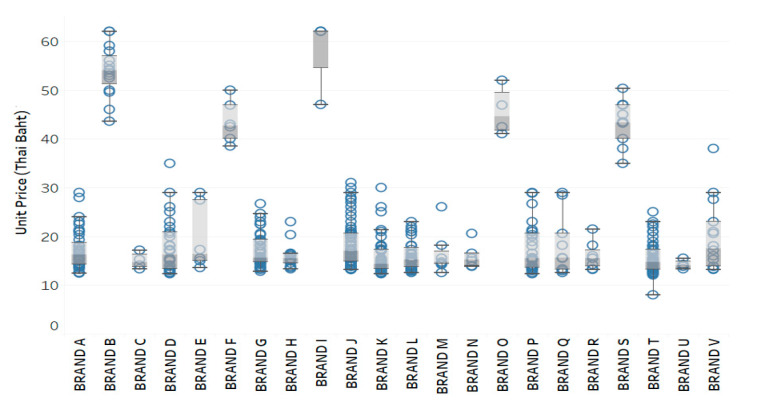
Example of distribution in drug prices across different brands of the omeprazole (40 mg) powder for solution for injection drug (ATC: A02BC01).

All medications listed on the procurement system in Thailand were classified according to the Anatomical Therapeutic Chemical Classification (ATC) system [38]. The drugs were defined by the chemical substance at the lowest level of the ATC code (5th level). In our analysis, we used medicine code A02BC01 (omeprazole, parenteral form) that presented high price dispersions. The dataset comprised 2424 records from January to March 2019. For this therapeutic category, we identified the mean, median, minimum, and maximum of unit prices according to the description of medical products used in the study. We also calculated the coefficient of variation (CV) to measure the relative dispersion of drug prices [39], as shown in Appendix A. We observed that there was high price dispersion for some medicine products, presenting an average CV exceeding 30%, which indicated that the purchasing process may out of control [40]. The price interval of each brand product of this medicine was represented in Figure 4.

The omeprazole 40 mg powder for solution for injection drug, resulting in a high range of unit price from 8.05 to 62.06 Thai Baht (THB). If we classified by trade names or brands of this drug as shown in Figure 4, the different product brands represented different price intervals. Some brands had high price dispersions. Some purchasers also bought it at high prices. This may vary in response to different other features, consisting of different trade product names with various pack sizes from different suppliers purchased by hospital purchasing managers in different health regions in Thailand with differing purchased budget volumes and procurement methods. These features were examined by using the feature selection method in the next section for removing irrelevant features and selecting only important features associated with the prediction of the prices for each medicine.

### 4.2. Feature Analysis

The choice of different methods of feature selections depends on both the algorithms being used and the type of given data. To examine whether the method of feature selection is suitable for the sequential minimal optimization (SMO) algorithm and pharmaceutical price data, we examined four popular feature selection methods implemented in the Waikato environment for knowledge analysis (WEKA) software, involving the correlation-based feature subset selection (CFS), information gain, gain ratio, and wrapper subset evaluation methods. Major features selected by these methods were ordered by the most relevant and compared to determine which of them can provide the greatest predictive accuracy. As presented in Table 2, the model’s accuracy rate was evaluated by using 10-fold cross-validation. The results indicated that the eight features selected by the gain ratio method provided the highest accuracy rate (92.62%), followed by those selected by the information gain, wrapper subset evaluation, and CFS methods (89.21%, 88.57%, and 84.15%, respectively). Accordingly, the model derived by using the SMO algorithm with the gain ratio method selected all the eight features, namely, the generic product groups, trade product name, dosage forms, suppliers, the segmented buyers, procurement methods, pack sizes, and total purchase budgets for predicting the range of drug prices and for providing the result at the highest predictive accuracy.

**Figure 5:**
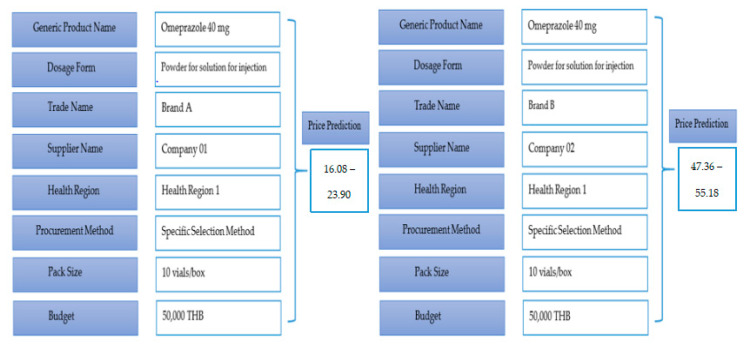
Examples of price prediction by using the model derived by the SMO algorithm with selection technique of the gain-ratio feature.

The work can be useful for health authorities or policymakers to estimate the prices of each medicine based on their procurement conditions. As described in Figure 5, for example, if it was necessary to predict the range of the prices for each vial of the omeprazole (40 mg) powder for solution for injection drug, the proposed model can estimate the range of the prices for a given set of the eight feature inputs. By comparison with previous price data shown in Figure 4, our model can suggest the smaller range of the purchase price of both brand A and brand B for hospital purchasing managers in the same health region in Thailand. This can control the distribution of the procurement price for the drug product.

In another work of this analysis, we can compare the prices for different sets of feature inputs such as comparing between brand A and brand B from different suppliers on the same pack size at 10 vials/box that will be procured by hospitals in the public health region 1 by using the specific selection procedure under the budget of 50,000 THB. The proposed model can predict the prices of both option inputs in a different range of prices. The price of brand A was about 3 times less than that of brand B. Thus, policymakers can monitor and analyze the prices of medicine from different options of feature inputs and provide this information to suggest the best choice for hospital purchasing managers in order to get the right medicine product at the optimal price.

### 4.3. Performance Evaluation

In our feature analysis, we used all eight condition features in developing the model for classifying the price of medicine corresponding to the eight class labels described in Figure 1. Our model provided good performance at an accuracy of approximately 92.62%, with high sensitivity and precision. Table 3 and Table 4 presented the evaluation results obtained after testing the algorithm using 10-fold cross-validation.

### 4.4. Confusion Matrix

**Figure 6:**
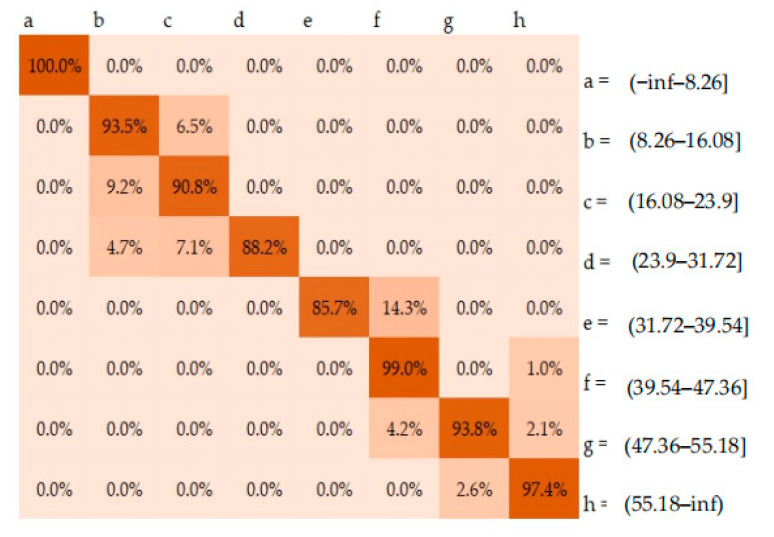
Normalized confusion matrix.

The decisions made by the classification model over a set of input data could be presented in the form of a confusion matrix, with each entry representing the numbers of both correct and incorrect predictions. This study involved a classification problem with eight classes, corresponding to an 8 × 8 confusion matrix, as shown in Figure 6. This represented visualization of the model performance, resulting in the proportion of correct predictions ranged from 0.857 to 1.

## 5. Discussion and Concluding Remarks

It is important to control the prices of medicine products and expenditure in the pharmaceutical sectors. Policymakers in many countries are currently considering and discussing a range of approaches intended to reduce drug prices and enhance the best options in the pharmaceutical marketplace. Pharmaceutical procurement data over time contribute useful insights when evaluating the need for new approaches and planning the policies [41]. However, measuring the change of the price for each medicine could be difficult because the market for pharmaceuticals is inefficient and complex [42]. In order to help policymakers to monitor the situations of medicine prices and provide feedbacks to obtain the best option for hospital purchasing managers to get good medicines with good prices effectively, this study aimed to investigate the performance of the model derived by the sequential minimal optimization (SMO) algorithm for predicting the range of drug prices on the procurement system.

SMO is one of the most widely used optimization algorithms among all effective algorithms in supporting vector learnings [24]. Platt demonstrated that SMO has greatly performed on scaling for multi-class classification [33]. It can perform with sparse data inputs, and is good even for imbalanced data. Therefore, the SMO algorithms should be explored for investigating their performance in predicting the range of the prices for each medicine in pharmaceutical procurement tasks that consisted of enormous features and different conditions. To do so, we considered three main hypotheses in this study. The first was that, among various features on the drug procurement data, we hypothesized that there were associations between the drug prices and the eight features of the procurement system, including the generic product names, trade product names, procurement methods, dosage forms, pack sizes, manufacturers, the segmented buyers, and total purchase budgets. These features can be adequate relevant data inputs used in developing the model to effectively predict the range of the prices for each medicine. The second hypothesis was that using SMO with a suitable feature selection method could improve the accuracy of prediction. This is because SMO is a classification approach that cannot obtain the relevant features directly. It requires feature selection tools for improving the predictive accuracy [43,44]. Currently, there are many techniques for statistical feature selection that can be used with SMO in many types of datasets; however, as far as we know, there is no study using this approach in the field of pharmaceuticals. Thus, our study bridged the gap in the literature to use feature selection techniques with the SMO algorithm and pharmaceutical data. We explored four popular feature selection techniques: correlation-based feature subset selection (CFS), information gain, gain ratio, and wrapper subset evaluator. The techniques were used to determine which one could provide the best predictive accuracy for the SMO model. Furthermore, in the context of developing a prediction model, the efficacy and benefits of using the SMO algorithm are reported. Therefore, the last hypothesis was that the model derived by the SMO algorithm could have a great performance for predicting the range of the prices for each medicine. This model performance was measured in terms of accuracy rate [45,46], Kappa coefficient, precision, recall (sensitivity), F-measure, receiver operating characteristic (ROC) area [47], and confusion matrix [48]. In the validation, we used the method of 10-fold cross-validation which was an extensive option for evaluating the performance of the classification model [23]. The method can average the error estimation over ten trials to receive the total model effectiveness, reduce the bias, and it has significantly less variance because all the data were used in the validation set.

In this study, we employed the pharmaceutical price data listed on Thailand’s government procurement system. Although some approaches have been developed to measure the change of the price for each medicine in Thailand, there is still no proper technique to determine the appropriate price and to control the distribution of the procurement price for drug products. Presently, policymakers collect the procurement data of medicine and estimate a reference price or benchmark for each medicine. This benchmark is set as the maximum price of the medicine group which consists of the same generic name and dosage form. For example, if the reference price of the omeprazole 40 mg drug, injection form, is estimated to be 62.06 Thai Baht (THB), inferring that all trade product names of this type of medicine must be purchased at lower or equal 62.06 THB for each vial. However, in our study, we found that under the price regulation policy through reference prices, drug products of the same generic name can be procured at different prices and showed high price dispersion. Meanwhile, different brands of the same chemical substance were also purchased at different ranges of prices (see Figure 4). More importantly, we found that using only two features of the generic name and dosage form for classifying the prices of medicine cannot provide the best predictive accuracy. As illustrated in Table 2, the estimation of medicine prices was more accurate when all the eight relevant features can be used together. This fully supported our first hypothesis and pointed out that the drug pricing in the current market could be different for different brands, pack sizes, suppliers, segmented buyers, and purchase budgets. This can suggest that although the reference price policy was implemented in Thailand, there was a problem of the price dispersion in the same generic medications, which in turn generated some considerable effects on pharmaceutical expenditure. Acosta et al. [18] also revealed this phenomenon and they suggested that the reference price strategy has led to reduced transparency and accessibility of drug price information, for example, pharmaceutical suppliers tried to prevent buyers from knowing that the same drug product was sold at different prices in different areas but claimed to be of the same price. There were higher differences in prices for medicine in markets with a greater number of manufacturers, different trade names, different packaging, and different procurement conditions. Another problem was that the health authorities did not have a broad understanding of the current market prices. Some hospitals may choose the medicine product with an expensive possible price in the market because they thought that the price was not above the reference price given, and they had no incentive to find out the cheaper one. These factors had significant impacts on how well hospitals can effectively select the product price on various combination features. Accordingly, using the reference price method alone engenders difficulty in monitoring and calculating the price of each medicine product. Thus, determining the range of drug prices over setting the single reference price can develop an interpretation and be compatible with heterogeneous data. The purchaser can recognize the lowest possible price of each medicine in order to negotiate the price with the suppliers.

To improve predictive accuracy, we incorporated the SMO algorithm with feature selection tools. We found that our proposed model of SMO algorithm with gain ratio selection method outperforms other models by providing the highest accuracy rate, followed by the information gain, wrapper subset evaluation, and CFS methods. This can explain the second hypothesis; the gain ratio can be the best choice of feature selection method that was suitable for the SMO algorithm and pharmaceutical price data in our study. For measuring model performance for predicting the range of the prices for medicine products presented in Table 3 and Table 4, the proposed model has very good prediction accuracy, precision, sensitivity, and F-measure. Additionally, the model provided substantial agreement for the Kappa coefficient as clarified with the results in the normalized confusion matrix. Consequently, the excellent performance of the SMO algorithm was proved on the basis of ROC analysis. The results fully supported our third hypothesis; the SMO algorithm is a powerful optimization technique for classifying data and predicting the pharmaceutical product prices. More specifically, we found that drug prices predicted by the proposed model can be represented in a lower price range, and users can compare drug prices among different options of feature inputs, as depicted in Figure 5, to find out the best solution for their conditions. Applying this model enables a suitable approach to intervention development by considering a range of relevant features corresponding to price dispersion in the pharmaceutical procurement system.

Our proposed model has some limitations. First, our results were based on the accuracy of the procurement data and the procurement prices of some types of medicines procured in the given time period. Different types of medicines could yield different findings. Thus, academics and practitioners can use more data and more types of products to examine the algorithms and compare the performance of our proposed model with other algorithms. Moreover, there could be a challenge in class distribution when using enormous differential types of medicines with complex purchasing conditions. Therefore, future analysis could also investigate the relationship between class distributions and classification effectiveness.

## 6. Conclusions

In conclusion, the model derived using the SMO algorithm is useful for enhancing data analysis in pharmaceutical procurement tasks. This study explicitly demonstrated the benefits of using this approach. One of them is that the model has useful analytics, and is sensitive and dependable [49,50]. Another reason is that it can allow policymakers to conduct analysis with their specific feature inputs or relevant procurement conditions [12]. The model can distinguish the differences in the prices of medicines in the pharmaceutical market by using relevant features: the characteristics of medicine product [27], the competitive potential of manufacturers or suppliers [8], the region-based health services system [5], and the procurement conditions [29]. In this study, our proposed model can be used to predict the range of medicine prices for any set of eight feature inputs in the procurement system. The application can help policymakers to better understand the situations of the pharmaceutical market and monitor the distributions of the prices for each drug. Specifically, they can provide the price information to suggest hospital purchasing managers in selecting greater choices to get the best possible price of medicine instead of providing only the reference price. Developing an efficient application can increase transparency in pharmaceutical purchasing, can reduce corruption risks, and can build up the circle of sufficient information between the government data center and hospitals. This could save a lot of money if the hospital can choose the right quality product at the lowest price and can lead to a reduction in drug expenditure. This finding can also be used for other countries that have been facing the challenge of using reference price policy.

## Figures and Tables

**Table 1:** The features and the definitions of the dataset in our study.

Features	Descriptions
DEPT	Purchasing departments who purchase the medicines for hospitals
GPU	The name of generic product use in the database which involves the virtual therapeutic moiety and strength (e.g., Omeprazole 40 mg)
TPU	The name of trade product use or brand (e.g., Losec^®^, Omezole^®^)
METHOD	Procurement method (e.g., bidding method, specific selection method)
WINNER	Supplier who sells the medical product
UNIT	The dosage form of the drug product (e.g., powder for solution for injection)
SIZE	The number of units per pack (e.g., 14 or 28 tablets per box)
TOTAL	Purchase budget for each medical product (Thai Baht)
PRICE ^a^	Procurement price per unit (Thai Baht)

^a^ Output variable corresponding to the range of prices for pharmaceutical products.

**Table 2:** Selected features ordered by most relevant for different feature selection methods and corresponding accuracy.

Selection Methods	Selected Features	% Accuracy Measure
CFS	(1) GPU, (2) UNIT (3) DEPT	84.15%
Wrapper Subset Evaluator	(1) GPU, (2) UNIT (3) DEPT, (4) TOTAL, (5) SIZE	88.57%
Information Gain	(1) GPU, (2) UNIT (3) DEPT, (4) TOTAL, (5) SIZE (6) TPU, (7) WINNER	89.21%
Gain Raito	(1) GPU, (2) UNIT (3) DEPT, (4) TOTAL, (5) SIZE (6) TPU, (7) WINNER, (8) METHOD	92.62%

Note. CFS = Correlation-Based Feature Subset Selection, TPU = Trade Product Name, GPU = Generic Product Name, UNIT = Dosage Form, DEPT = The Segmented Buyers, WINNER = Manufacturer/Vendor, TOTAL = Total Purchase Budget, METHOD = Procurement Method, SIZE = The Number of Units Per Pack.

**Table 3:** Performance evaluation results.

Class Labels	*n* (%)	TP Rate	FP Rate	Precision	Recall	F-Measure	ROC Area
class 1 = (−inf–8.26]	5(0.2)	1.000	0.000	1.000	1.000	1.000	1.000
class 2 = (8.26–16.08]	1238(51.1)	0.935	0.067	0.935	0.935	0.935	0.943
class 3 = (16.08–23.9]	802(33.1)	0.908	0.055	0.891	0.908	0.899	0.935
class 4 = (23.9–31.72]	127(5.2)	0.882	0.000	1.000	0.882	0.937	0.978
class 5 = (31.72–39.54]	28(1.2)	0.857	0.000	1.000	0.857	0.923	0.974
class 6 = (39.54–47.36]	98(4.0)	0.990	0.003	0.942	0.990	0.965	0.990
class 7 = (47.36–55.18]	48(2.0)	0.938	0.001	0.957	0.938	0.947	0.977
class 8 = (55.18–inf)	78(3.2)	0.974	0.001	0.974	0.974	0.974	0.987
Weighted Average		0.926	0.053	0.927	0.926	0.926	0.947

Note. TP Rate = True Positive Rate, FP Rate = False positive rate, ROC Area = Receiver operating characteristic area.

**Table 4:** Summary results.

Parameters	Results
Correctly Classified Instances, *n* (%)	2245 (92.62%)
Incorrectly Classified Instances, *n* (%)	179 (7.38%)
Kappa statistic	0.8813
Mean absolute error	0.1883
Root mean squared error	0.2925
Total Number of Instances	2424

## Data Availability

Data are contained within the article.

## Appendix A

**Table A1:** Descriptive statistics of medicine code A02BC01 (omeprazole 40 mg, parenteral) used in this study.

Trade Names	*n* (%)	Median	Mean	Minimum	Maximum	% CV
BRAND A	278.0	16.1	17.1	12.4	29.0	16.9
BRAND B	117.0	56.0	55.1	43.6	62.1	10.0
BRAND C	4.0	14.7	15.0	13.4	17.1	10.9
BRAND D	56.0	17.1	18.4	12.4	35.0	35.6
BRAND E	8.0	15.2	18.2	13.7	29.0	35.1
BRAND F	24.0	42.4	42.9	38.5	50.0	7.4
BRAND G	255.0	15.3	16.6	12.8	26.8	17.4
BRAND H	19.0	16.2	17.0	13.4	23.0	18.7
BRAND I	15.0	62.1	61.1	47.1	62.1	6.3
BRAND J	719.0	18.0	18.8	13.2	31.0	22.5
BRAND K	195.0	13.2	14.4	12.4	30.0	21.6
BRAND L	68.0	15.3	15.9	12.5	23.0	18.7
BRAND M	12.0	15.5	18.7	12.5	26.0	29.5
BRAND N	10.0	15.0	15.4	13.9	20.7	13.0
BRAND O	22.0	46.9	46.7	41.0	52.0	5.2
BRAND P	50.0	15.5	17.8	12.4	29.0	28.0
BRAND Q	12.0	15.5	17.6	12.5	29.0	32.5
BRAND R	9.0	15.5	15.9	13.2	21.5	16.2
BRAND S	58.0	43.2	42.3	35.0	50.3	10.1
BRAND T	463.0	13.6	14.5	8.1	25.0	17.8
BRAND U	4.0	14.0	14.2	13.4	15.5	6.4
BRAND V	26.0	16.6	20.9	13.2	38.0	42.7

## References

[1] World Health Organization (WHO) WHO Guideline on Country Pharmaceutical Pricing Policies. https://www.who.int/publications/i/item/9789240011878.

[2] Gregson N., Sparrowhawk K., Mauskopf J., Paul J. (2005). Pricing medicines: Theory and practice, challenges and opportunities. Nat. Rev. Drug Discov..

[3] Das S.C., Mandal M., Mandal S.C. (2007). A critical study on availability and price variation between different brands: Impact on access to medicines. Ind. J. Pharm. Sci..

[4] Danzon P.M., Kim J.D. (1998). International price comparisons for pharmaceuticals: Measurement and policy issues. Pharmacoeconomics.

[5] Udomaksorn S., Sakulbumrungsil R.C., Luangruangrong P. (2008). The Investigation of Pharmaceutical Price Discrimination Among Public Hospitals in Thailand: A Case Study of Agent Acting on the Renin Angiotensin System (ACE) Inhibitors. Thai J. Pharm. Sci..

[6] Ngorsuraches S., Chaiyakan K. (2015). Equitable Prices of Single-Source Drugs in Thailand. Appl. Health Econ. Health Policy.

[7] Songthung P., Sripanidkulchai K., Luangruangrong P., Sakulbumrungsil R.C., Udomaksorn S., Kessomboon N., Kanchanaphibool I. An Innovative Decision Support Service for Improving Pharmaceutical Acquisition Capabilities. Proceedings of the 2012 Annual SRII Global Conference.

[8] Sooksriwong C., Suwattanapreeda S., Chanjaruporn F. (2009). Medicine prices in Thailand: A result of no medicine pricing policy. South Med. Rev..

[9] Jung S., Bi Y., Davuluri R.V. (2015). Evaluation of data discretization methods to derive platform independent isoform expression signatures for multi-class tumor subtyping. BMC Genom..

[10] Sidey-Gibbons J.A.M., Sidey-Gibbons C.J. (2019). Machine learning in medicine: A practical introduction. BMC Med. Res. Methodol..

[11] Platt J.C., Kearns M.S., Solla S.A., Cohn D.A. (1999). Using sparseness and analytic QP to speed training of support vector machines. Advances in Neural Information Processing Systems 1999.

[12] Naveed H., Khan G., Khan A.U., Siddiqi A., Khan M.U.G. (2019). Human activity recognition using mixture of heterogeneous features and sequential minimal optimization. Int. J. Mach. Learn Cybern..

[13] Seidman G., Atun R. (2017). Do changes to supply chains and procurement processes yield cost savings and improve availability of pharmaceuticals, vaccines or health products? A systematic review of evidence from low-income and middle-income countries. BMJ Glob. Health.

[14] Kohler J.C., Dimancesco D. (2020). The risk of corruption in public pharmaceutical procurement: How anti-corruption, transparency and accountability measures may reduce this risk. Glob. Health Action.

[15] Ioannides-Demos L.L., Ibrahim J.E., McNeil J.J. (2002). Reference-Based Pricing Schemes. Pharmacoeconomics.

[16] Acosta A., Ciapponi A., Aaserud M., Vietto V., Austvoll-Dahlgren A., Kösters J.P., Vacca C., Machado M., Diaz Ayala D.H., Oxman A.D. (2014). Pharmaceutical policies: Effects of reference pricing, other pricing, and purchasing policies. Cochrane Database Syst. Rev..

[17] Andia T., Gaviria A., Gomez C., Jaramillo L.F., Marquez S., Rodríguez I., Vaca C. (2014). First Evaluation of Colombian’s External Reference Pricing System. Value Health.

[18] Acosta A., Basto S., Fonseca M.F., Duran C., Vargas C., Rovira J. (2018). Description of Drug Pricing and Procurement Information Web Portals in Some Latin American Countries. Pharmacoeconomics.

[19] Chen L., Yang Y., Luo M., Hu B., Yin S., Mao Z. (2020). The Impacts of National Centralized Drug Procurement Policy on Drug Utilization and Drug Expenditures: The Case of Shenzhen, China. Int. J. Environ. Res. Public Health.

[20] Dinov I.D. (2018). Data Science and Predictive Analytics: Biomedical and Health Applications Using R.

[21] Roumani Y.F., May J.H., Strum D.P., Vargas L.G. (2012). Classifying highly imbalanced ICU data. Health Care Manag. Sci..

[22] Keerthi S.S., Shevade S.K., Bhattacharyya C., Murthy K.R.K. (2001). Improvements to Platt’s SMO Algorithm for SVM Classifier Design. Neural. Comput..

[23] Pham B.T., Tien Bui D., Prakash I., Nguyen L.H., Dholakia M.B. (2017). A comparative study of sequential minimal optimization-based support vector machines, vote feature intervals, and logistic regression in landslide susceptibility assessment using GIS. Environ. Earth Sci..

[24] Mohammed S.A., Darrab S., Noaman S.A., Saake G., Tan Y., Shi Y., Tuba M. (2020). Analysis of Breast Cancer Detection Using Different Machine Learning Techniques. Data Mining and Big Data. Communications in Computer and Information Science.

[25] Sunarya A., Henderi H., Tasyriqan I. The comparison between sequential minimal optimization and multilayer perceptron neural network methods in predicting the commodity prices. Proceedings of the 2019 Fourth International Conference on Informatics and Computing (ICIC).

[26] Ince H., Trafalis T.B. (2008). Short term forecasting with support vector machines and application to stock price prediction. Int. J. Gen. Syst..

[27] Kwon S.H., Park H.S., Na Y.J., Park C., Shin J.Y., Kim H.L. (2020). Price Reduction of Anticancer Drugs from 2007 to 2019 in South Korea: The Impact of Pharmaceutical Cost-Containment Policies. Appl. Health Econ. Health Policy.

[28] Ongarora D., Karumbi J., Minnaard W., Abuga K., Okungu V., Kibwage I. (2019). Medicine prices, availability, and affordability in private health facilities in low-income settlements in Nairobi County, Kenya. Pharmacy.

[29] Kwon H.Y., Godman B. (2017). Drug Pricing in South Korea. Appl. Health Econ. Health Policy.

[30] Emanuel E., Tanden N., Altman S., Armstrong S., Berwick D., Brantes F., Calsyn M., Chernew M., Colmers J., Cutler D. (2012). A systemic approach to containing health care spending. N. Engl. J. Med..

[31] Saleh B., Saedi A., Aqbi A., Salman L. (2020). Analysis of Weka Data Mining Techniques for Heart Disease Prediction System. Int. J. Med. Rev..

[32] Patil P.C., Panchal P.S., Madiwale S., Tale V.S. (2020). An analysis of non-cultivable bacteria using WEKA. Bioinformation.

[33] Platt J.C. (1999). Fast training of support vector machines using sequential minimal optimization. Advances in Kernel Methods: Support Vector Learning.

[34] Yao W.F., Jia X.B. (2014). An Improved SVM Based on Feature Extension and Feature Selection. Appl. Mech. Mater..

[35] Roobaert D., Karakoulas G., Chawla N.V., Guyon I., Nikravesh M., Gunn S., Zadeh L.A. (2006). Information gain, correlation and support vector machines. Feature Extraction. Studies in Fuzziness and Soft Computing.

[36] Liu Y., Bi J.W., Fan Z.P. (2017). Multi-class sentiment classification: The experimental comparisons of feature selection and machine learning algorithms. Expert Syst. Appl..

[37] Sokolova M., Lapalme G. (2009). A systematic analysis of performance measures for classification tasks. Inf. Process Manag..

[38] MacDonald K., Potvin K. (2004). Interprovincial Variation in Access to Publicly Funded Pharmaceuticals. Can. Pharm. J..

[39] Bernstein J.J.J., Holt G.B., Bernstein J. (2019). Price dispersion of generic medications. PLoS ONE.

[40] Brown C.E. (2017). Coefficient of Variation. Applied Multivariate Statistics in Geohydrology and Related Sciences.

[41] Hamzah N.M., Perera P.N., Rannan-Eliya R.P. (2020). How well does Malaysia achieve value for money in public sector purchasing of medicines? Evidence from medicines procurement prices from 2010 to 2014. BMC Health Serv. Res..

[42] Suchonwanich N., Laowahutannon T., Luangruangrong P., Techathawat S., Wongtangprasert S. (2020). Drug Procurement and Distribution. J. Health Sci..

[43] Al-Shahib A., Breitling R., Gilbert D. (2005). Feature Selection and the Class Imbalance Problem in Predicting Protein Function from Sequence. Appl. Bioinform..

[44] Gao L., Ye M., Lu X., Huang D. (2017). Hybrid Method Based on Information Gain and Support Vector Machine for Gene Selection in Cancer Classification. Genom. Proteom. Bioinform..

[45] Araújo F.H.D., Santana A.M., Santos Neto P. (2016). Using machine learning to support healthcare professionals in making preauthorisation decisions. Int. J. Med. Inform..

[46] Jiao Y., Du P. (2016). Performance measures in evaluating machine learning based bioinformatics predictors for classifications. Quant. Biol..

[47] Fawcett T. (2006). An introduction to ROC analysis. Pattern. Recognit. Lett..

[48] Luque A., Carrasco A., Martín A., Heras A. (2019). The impact of class imbalance in classification performance metrics based on the binary confusion matrix. Pattern. Recognit..

[49] Hu Y.C., Ansell J. (2009). Retail default prediction by using sequential minimal optimization technique. J. Forecast..

[50] Zhao Z., Li X.Y. (2013). Study of Sequential Minimal Optimization Algorithm Type and Kernel Function Selection for Short-Term Load Forecasting. Appl. Mech. Mater..

